# Supplementary material for: Lnc-uc.147 Is Associated with Disease Stage of Liver, Gastric, and Renal Cancer
Source: Biomolecules. 2023 Jan 31;13(2):265. doi: 10.3390/biom13020265 (PMC9953473; doi:10.3390/biom13020265)
Supplement: Supplementary file 1 [file biomolecules-13-00265-s001.zip › biomolecules-2068405-supplementary.pdf]

Supplementary Material

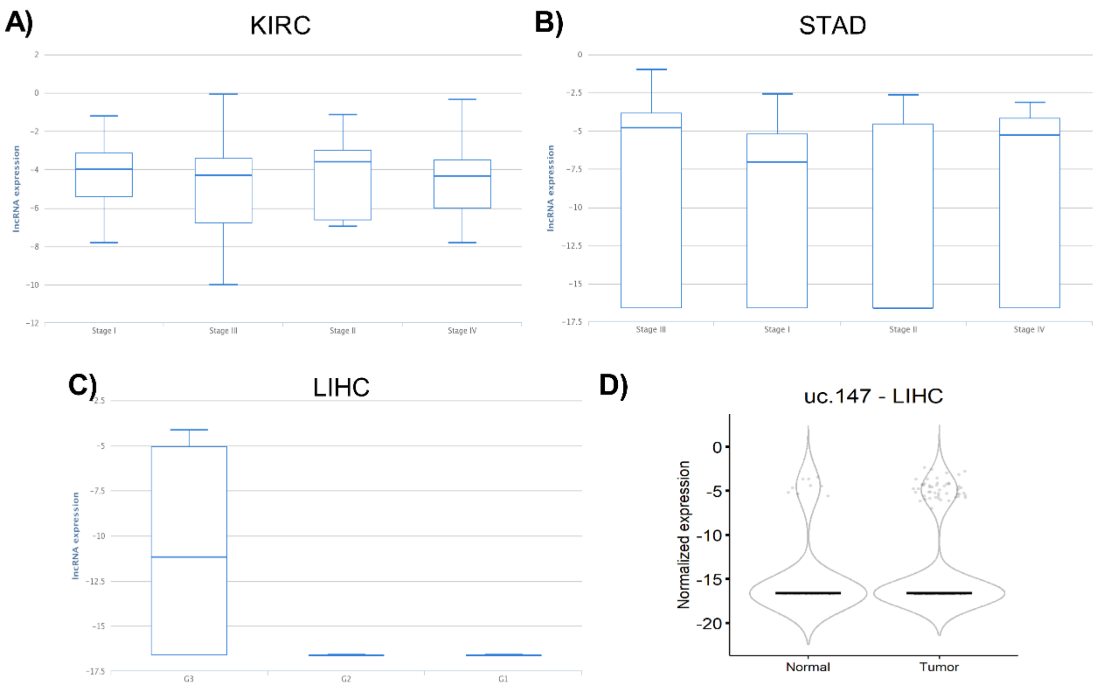

Figure S1 – Associations of lnc-uc.147 expression with kidney renal cell carcinoma (KIRC – A), stomach adenocarcinoma (STAD – B), and hepatocellular carcinoma (LIHC – C). Graphs were generated by the TANRIC platform (https://www.tanric.org/), and the *p*-values associated with this analysis are presented in Table 1. D) Boxplot demonstrating the expression pattern of lnc-uc.147 in non-tumoral and tumoral samples for LIHC. Through Mann-Whitney analysis, no difference between the two groups is observed.
